# Reduced Phrenic Motoneuron Recruitment during Sustained Inspiratory Threshold Loading Compared to Single-Breath Loading: A Twitch Interpolation Study

**DOI:** 10.3389/fphys.2016.00537

**Published:** 2016-11-10

**Authors:** Mathieu Raux, Alexandre Demoule, Stefania Redolfi, Capucine Morelot-Panzini, Thomas Similowski

**Affiliations:** ^1^Sorbonne Universités, UPMC - University Pierre and Marie Curie Univ Paris 06, Institut National de la Santé et de la Recherche Médicale, UMRS1158 Neurophysiologie Respiratoire Expérimentale et cliniqueParis, France; ^2^AP-HP, Groupe Hospitalier Pitié-Salpêtrière Charles Foix, Département d'Anesthésie-RéanimationParis, France; ^3^AP-HP, Groupe Hospitalier Pitié-Salpêtrière Charles Foix, Service de Pneumologie et Réanimation Médicale (Département“R3S”)Paris, France; ^4^AP-HP, Groupe Hospitalier Pitié-Salpêtrière Charles Foix, Service des Pathologies du Sommeil (Département “R3S”)Paris, France

**Keywords:** control of breathing, cerebral cortex, diaphragm, twitch interpolation, inspiratory loading

## Abstract

In humans, inspiratory constraints engage cortical networks involving the supplementary motor area. Functional magnetic resonance imaging (fMRI) shows that the spread and intensity of the corresponding respiratory-related cortical activation dramatically decrease when a discrete load becomes sustained. This has been interpreted as reflecting motor cortical reorganization and automatisation, but could proceed from sensory and/or affective habituation. To corroborate the existence of motor reorganization between single-breath and sustained inspiratory loading (namely changes in motor neurones recruitment), we conducted a diaphragm twitch interpolation study based on the hypothesis that motor reorganization should result in changes in the twitch interpolation slope. Fourteen healthy subjects (age: 21–40 years) were studied. Bilateral phrenic stimulation was delivered at rest, upon prepared and targeted voluntary inspiratory efforts (“vol”), upon unprepared inspiratory efforts against a single-breath inspiratory threshold load (“single-breath”), and upon sustained inspiratory efforts against the same type of load (“continuous”). The slope of the relationship between diaphragm twitch transdiaphragmatic pressure and the underlying transdiaphragmatic pressure was −1.1 ± 0.2 during “vol,” −1.5 ± 0.7 during “single-breath,” and −0.6 ± 0.4 during “continuous” (all slopes expressed in percent of baseline.percent of baseline^−1^) all comparisons significant at the 5% level. The contribution of the diaphragm to inspiration, as assessed by the gastric pressure to transdiaphragmatic pressure ratio, was 31 ± 17% during “vol,” 22 ± 16% during “single-breath” (*p* = 0.13), and 19 ± 9% during “continuous” (*p* = 0.0015 vs. “vol”). This study shows that the relationship between the amplitude of the transdiaphragmatic pressure produced by a diaphragm twitch and its counterpart produced by the underlying diaphragm contraction is not unequivocal. If twitch interpolation is interpreted as reflecting motoneuron recruitment, this study supports motor reorganization compatible with “diaphragm sparing” when an inspiratory threshold load becomes sustained.

## Introduction

In humans, the net neural drive that is conveyed from the central nervous system to the respiratory muscles results from the integration of multiple inputs by respiratory spinal motoneurons (review in Butler et al., 2014). Permanent phasic descending motor inputs are derived from brainstem central pattern generators (bulbospinal drive). Cortical and cortico-subcortical circuits project onto respiratory spinal motoneurons (corticospinal drive). They account for voluntary ventilatory capabilities and tonically contribute to the so-called “wakefulness drive to breathe” (Laviolette et al., 2013; Tremoureux et al., 2014; Dubois et al., 2016). Cortical and cortico-subcortical circuits with respiratory projections are also engaged during inspiratory load compensation (Raux et al., 2007a,b, 2013). Electroencephalographic data have evidenced premotor cortical potentials preceding inspiration (pre-inspiratory potentials) in normal individuals exposed to inspiratory constraints (Raux et al., 2007a,b). Such potentials constitute a signature of cortical activity originating in the supplementary motor complex, which includes the supplementary motor area (SMA) (Ball et al., 1999; Shibasaki, 2012). Functional magnetic resonance imaging data (fMRI) have confirmed that inspiratory threshold loading induces complex cortical activation (Raux et al., 2013), and have shown that the spread and intensity of the corresponding respiratory-related cortical activation dramatically decrease when a discrete load (presented to the subject on a single breath basis) becomes sustained (Raux et al., 2013). These reductions in cortical activation are observed both in premotor/motor and sensory cortical areas (Raux et al., 2013). They have been interpreted as reflecting motor cortical reorganization and automatization (Raux et al., 2013). They are also likely to proceed from sensory and/or affective habituation (von Leupoldt et al., 2009), that could modify not only activies in sensory areas but also in premotor/motor areas through cortico-cortical connections. fMRI therefore does not give direct indications about the changes in the central motor drive to respiratory spinal motoneurons that occur when inspiratory loading becomes sustained. This is however a relevant issue in view of the relationship that exist between the neural drive to breathe, respiratory muscle fatigue, and dyspnea (Marin et al., 1999; Reilly et al., 2011; Suh et al., 2015; Georges et al., 2016) and the importance of avoiding diaphragm fatigue. In this regard, it has been suggested that inspiratory threshold loading can be associated with “diaphragm sparing” mechanisms in certain circumstances (Jonville et al., 1985).

The neural drive to the diaphragm can be estimated by taking advantage of the twitch interpolation phenomenon (Merton, 1954; Bellemare and Bigland-Ritchie, 1984; Gandevia, 2001), as follows. The amplitude of the response of a muscle to supramaximal stimulation of its parent nerve decreases linearly with the intensity of an underlying contraction (Merton, 1954; Gandevia, 2001). This decrease depends on the proportion of the motoneurons pool that is recruited by the underlying contraction. The slope of the relationship between the underlying effort and the twitch response is therefore a reflection of the motoneurons recruitment dynamics. It is not dependent on any sensory factor. We designed the present study to corroborate the fMRI-derived contention that at least part of the difference in cortical activity between sustained inspiratory threshold loading and single-breath loading corresponds to motor reorganization, and to provide quantitative estimates of this reorganization. We hypothesized that changes in the intensity and/or organization of the corticospinal outflow to phrenic motoneurons with sustained inspiratory loading should result in changes in the slope slope of the relationship between the underlying effort and the diaphragm twitch response. We therefore compared the pattern of the diaphragm twitch interpolation induced by unprepared inspiratory efforts in response to unexpected single-breath threshold loads of varying intensity and by sustained efforts against the same of type of loads. The twitch interpolation pattern produced by voluntary, well prepared, and precisely targeted diaphragmatic contractions served as a reference.

## Material and methods

### Subjects

Fourteen healthy subjects (age: 21–40 years, 6 men and 8 women, BMI: 18–25 kg.m^−2^) participated in the study after ethical clearance from the appropriate external authority (*CPPRB Pitié-Salpêtrière, Paris, France*). All subjects received detailed information regarding the purpose of the study and methods used, and gave their written consent to participate.

### Experimental setup

All subjects were studied while sitting in a comfortable rest chair, with the abdomen unbound. They wore a nose clip and breathed through a mouthpiece in a non-rebreathing valve (Hans-Rudolf 2600, Kansas City, MO, USA) of which the inspiratory limb could be silently occluded by an inflatable balloon (Hans-Rudolf 9340 occlusion valve and 9330 compressor, Kansas City, MO, USA).

### Measurements

#### Electromyograms (EMG)

Surface recordings of the left and right costal diaphragmatic electromyographic activities were obtained using two pairs of skin-taped silver cup electrodes placed in the 7th right and left intercostal spaces, according to the technique described by Verin et al. (2002). These electrodes were connected to a Neuropack Sigma electromyograph (Nihon Kohden, Tokyo, Japan). Signals were amplified and band-pass filtered (20–3000 Hz) then sampled at 4 kHz and digitized (Chart v4.2, AD Instruments, Castle Hill, Australia) to be stored on an Apple Macintosh G4 Computer for off-line analysis.

#### Pressures

Oesophageal and gastric pressures (Pes and Pga) were measured according to standard practice (American Thoracic Society European Respiratory Society ATS/ER, 2002) using two balloon catheters (C76080U 1.9 mm × 80 mm, ID 1.4 mm, Marquat Gbm, Boissy-St-Léger, France) inserted through the nose after topical anesthesia (5% lidocaine). They were connected to two separate Validyne DP-15 differential pressure transducers (±200 cm H_2_O; Validyne, Northridge, CA, USA). Transdiaphragmatic pressure (Pdi) was obtained on-line by deriving Pes and Pga from a third transducer. The pressure signals were sampled at 400 Hz and digitized. The Pdi trace could be displayed to the subjects.

#### Abdominal displacements

Abdominal displacements were recorded using a belt-mounted strain gauge placed just above the umbilicus (Sleepmate, Resp-EZ, Midlothian, VA, USA).

#### Phrenic stimulation

Bilateral phrenic stimulation was delivered by cervical magnetic stimulation (CMS), using a Magstim 200 stimulator (The Magstim Co., Sheffield, UK) equipped with a 90 mm circular coil (2.5 Tesla, 0.1 ms square-wave pulses) placed over the 7th cervical spinal process (Similowski et al., 1989), and triggered either manually or automatically from the Pdi signal. The supramaximal nature of stimulation was determined by simplified recruitment curves constructed in terms of both the EMG and Pga responses to CMS.

### Procedures

#### Static pressures

Maximal inspiratory pressures (Pi,max) were measured according to standard practice (American Thoracic Society European Respiratory Society ATS/ER, 2002). American Thoracic Society European Respiratory Society ATS/ER (2002). Maximal transdiaphragmatic pressure (Pdi,max) was measured against an occluded airway using a combined expulsive-Mueller maneuvre with visual feedback according to Laporta and Grassino (1985). Pdi,max was defined as the value achieved during the best of three consecutive attempts differing by less than 20%.

#### Relaxed diaphragmatic twitches

Relaxed diaphragmatic twitches in response to supramaximal CMS were obtained immediately after the static maneuvres described above. The baseline reference values reported in this study therefore correspond to potentiated twitches (Mador et al., 1994; Gandevia, 2001)

#### Twitch interpolation experiments

In a first experiment, phrenic stimulation was superimposed upon prepared and targeted voluntary inspiratory efforts of various magnitude (“vol” procedure). The subjects were asked to produce voluntary inspirations from their functional residual capacity (FRC) against an occluded airway (Bellemare and Bigland-Ritchie, 1984). The Pdi trace was displayed on an oscilloscope, and the Pdi value set to trigger the stimulator (Pdi,vol) was indicated on the screen. The subjects were instructed to target this value and were informed that this would trigger the stimulation. No further instructions were provided. Trigger levels ranged from 5 to 80% of Pdi,max (see discussion). Three stimulations were applied at each trigger level.

In a second experiment, phrenic stimulation was delivered at various Pdi levels produced by nonprepared, nontargeted, inspiratory efforts, on a single breath basis (“single-breath” procedure. For this purpose, the inspiratory limb of the respiratory valve was randomly and unexpectedly occluded during the previous expiration. This occlusion therefore confronted the subjects with an infinite resistance at the start of the next inspiration. During this occluded inspiration, the Pdi signal triggered the stimulation when it reached the pre-set trigger level. No Pdi feedback was provided, and the subjects were unaware of the pre-set trigger level. Three stimulations were applied at each trigger level. Trigger levels ranged from 5 to 80% of Pdi,max. Occlusion of the inspiratory port was removed during the immediate subsequent expiration.

In a third experiment, phrenic nerve stimulation was delivered during the tenth to fifteenth inspirations following the sustained presentation of an added adjustable inspiratory threshold load (“continuous” procedure) ranging from 5 to a maximum of 45 cm H_2_O in order to avoid excessive breathing discomfort. Phrenic stimulation was triggered by the Pdi signal. No Pdi feedback was provided to the subjects, who where unaware of the pre-set trigger level. Three stimulations were applied at each trigger level.

### Data analysis

#### Quality criteria

**Figure 1:**
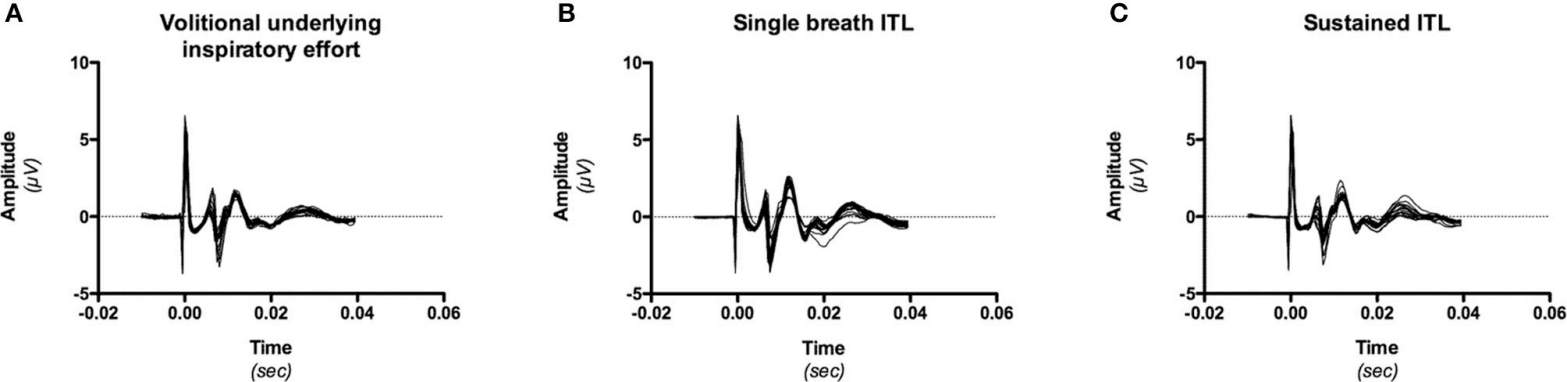
**Example, in one subject (# 9) of M-wave consistency during the three experimental conditions** [volitional underlying effort, **(A)**; single breath inspiratory threshold loading (ITL), **(B)**; sustained inspiratory threshold loading (ITL), **(C)**]. M-wave could differ in shape and amplitude between conditions (in line with known effects of lung volume and thoracoabdominal configuration), but were consistent within a given conditon (see “data management” in “Methods,” 2.5.2).

Data were analyzed offline using Chart software (Chart v4.2, AD Instruments, Castle Hill, Australia). Diaphragm twitches were considered suitable for analysis only if (1) they were obtained at relaxed FRC as determined from the Pes and abdominal displacement traces immediately preceding the stimulation (relaxed twitches) or the beginning of the corresponding inspiratory effort (superimposed twitches); (2) they were associated with an increase in abdominal circumference; and (3) they corresponded to an electromyographic M-wave amplitude differing by less than 15% from the maximal amplitude identified by the recruitment curves (Figure 1).

#### Data management

The amplitudes of the twitch pressures were measured from baseline to peak. The amplitudes of the diaphragmatic EMG were measured from peak to peak. The triggering values of Pdi were expressed as a percentage of the measured Pdi,max. To account for between-subject differences in anthropometric characteristics and muscle strength, the interpolated Pdi,tw were expressed as a percentage of the maximal Pdi,tw observed at any time during the procedure. In most subjects, the maximal Pdi,tw (used for normalization purposes) was greater than the baseline Pdi,tw value obtained from the relaxed diaphragm and was generally obtained with a slight underlying inspiratory effort (10–20 cm H_2_O), probably in line with changes in abdominal geometry and compliance between the relaxed and the active condition. Such changes are liable to modify the pressure generated by a diaphragm contraction of identical strength (Chen et al., 2000; Hershenson et al., 1988). For the same reason, the baseline Pdi,tw values were not taken into account to compute the twitch interpolation relationship. Pdi,tw values equal to zero were also excluded. The contribution of the diaphragm to the underlying effort was studied in terms of the Pga/Pdi ratio (Gilbert et al., 1981).

#### Statistics

After verifying normality and homoscedasticity, the results were expressed as mean ± SD, and comparisons were performed using analysis of variance for repeated measures with the *post-hoc* PLSD test or the Student *t*-test when appropriate (Winer et al., 1991). Y-intercepts of linear regressions were compared to 100% using the univariate sign test. Differences were considered significant when the risk of a type I error was less than 5% (*p* <0.05).

## Results

Static Pi,max, Pdi,max, and baseline Pdi,tw were within the normal range in all subjects (Table 1).

**Table 1:** **Subject characteristics and inspiratory pressure values**.

**Subject**	**Gender**	**Age *(years)***	**Height *(cm)***	**Weight *(Kg)***	**Pi,max *(cm H2O)***	**Pdi,max *(cm H2O)***	**Pdi,tw *(cm H2O)***
1	M	31	180	68	123	139	15
2	M	33	175	76	117	158	20
3	F	33	170	68	72	152	35
4	F	28	164	63	49	125	24
5	F	24	167	49	81	127	21
6	M	28	195	94	117	136	21
7	F	27	170	60	95	141	22.5
8	M	30	173	65	129	146	43.7
9	F	40	172	57	77	112	18
10	M	25	180	90	141	198	NA
11	M	27	185	68	80	125	28.4
12	F	25	163	55	87	166	26.8
13	F	27	175	54	NA	116	23.6
14	F	24	180	60	NA	126	20.4
mean		28.71	174.93	66.21	97.33	140.50	24.57
SD		4.41	8.62	12.97	27.65	22.76	7.63

*Pi,max, maximal static inspiratory pressure, Pdi,max, maximal transdiaphragmatic pressure measured during static manoeuvres, Pdi,tw, twitch transdiaphragmatic pressure elicited by cervical magnetic stimulation with the diaphragm relaxed (potentiated twitches). All data were obtained according to ATS-ERS recommendations (American Thoracic Society European Respiratory Society ATS/ER, 2002). American Thoracic Society European Respiratory Society ATS/ER (2002) at functional residual capacity*.

### Volitional twitch interpolation (“vol” procedure)

**Figure 2:**
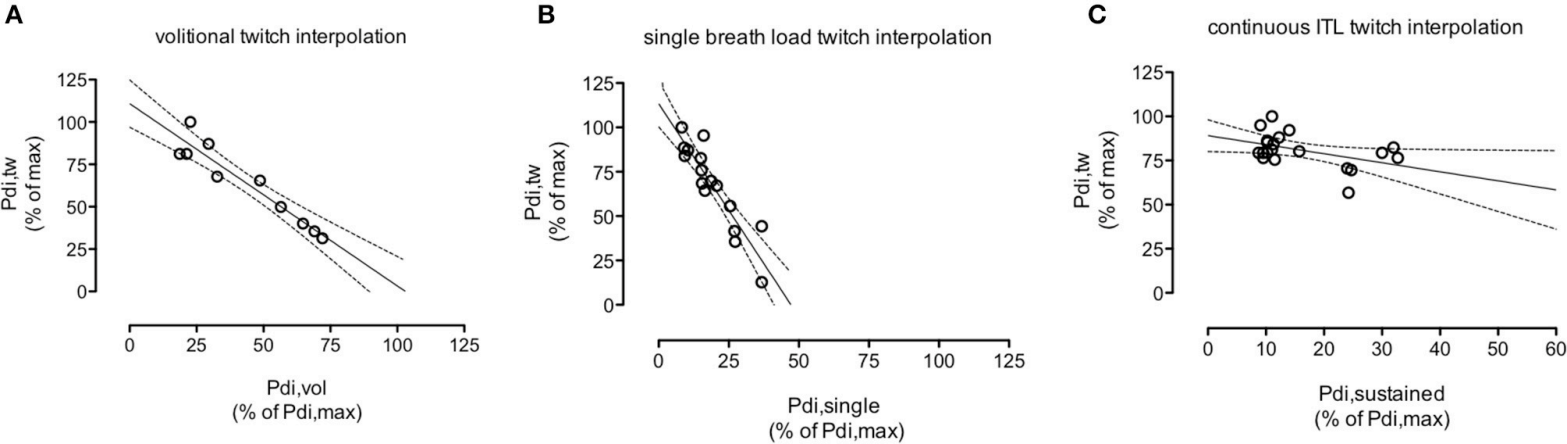
**Example, in one subject (#4), of the course of the twitch transdiaphragmatic pressure (Pdi,tw) elicited by cervical magnetic stimulation as a function of the intensity of the underlying diaphragm contraction. (A):** voluntary effort; **(B)**: non-volitional “single-breath” effort in response to the first presentation of an infinite resistance. **(C)**: non-volitional “continuous” effort, 10 breathing cycles after application of an inspiratory threshold load (ITL). The intensity of the underlying diaphragmatic contractions (abscissa) is expressed as a percentage of the maximal transdiaphragmatic pressure measured by static manoeuvres (Pdi,max). Pdi,tw (ordinate) are expressed as a percentage of their maximal values measured during the procedure considered. Solid lines depict the linear regression, dotted lines depict the corresponding 95% confidence interval. Note that the “continuous ITL” scales differ from the other scales because of the threshold load range (0 to 45 cm H_2_O). In the subject that this figure depicts, panel B shows that a diaphragm contraction producing a Pdi amounting to about 50% of the maximal Pdi recorded during voluntary contractions corresponded to near maximal diaphragm activation (phrenic stimulation triggered by higher pressure did not produce further pressure increments). In other words, the recruitment of phrenic motoneurons required to produce a given pressure was greater during the “single breath” procedure than during the voluntary procedure. During the “continuous ITL procedure” **(C)**, we needed the subjects to be able to breathe in sustainedly, and therefore the inspiratory limb of the breathing circuit could not be occluded. We chose inspiratory threshold values in a reasonable range regarding the induced respiratory discomfort. What **(C)** shows is that the Pdi,tw value corresponding to about 35% of Pdi,max is twice higher than the Pdi,tw value corresponding to the same percentage of Pdi,max during the “single breath” procedure **(B)**. This supports our hypothesis of a differrent dynamica of phrenic motoneurons recruitment between single breath loading and sustained loading.

Over the range of voluntary contraction force studied, Pdi,tw decreased linearly with the intensity of the underlying volitional inspiratory efforts (Figure 2A) according to Pdi,tw = b–a x Pdi,vol (Equation 1). With Pdi,tw expressed as a percentage of the maximal value observed during the procedure and Pdi,vol expressed as a percentage of Pdi,max (see methods), the slope “a” of Equation 1 was −1.1 ± 0.2 percent baseline.percent baseline^−1^ and its Y-intercept “b” was 100 ± 12%, not significantly different from 100% (*p* = 0.42). The extrapolation of Equation 1 through the abscissa yielded a “predicted” Pdi,max (Pdi,max,vol) that was not significantly different from the measured Pdi,max (*p* = 0.89).

**Figure 3:**
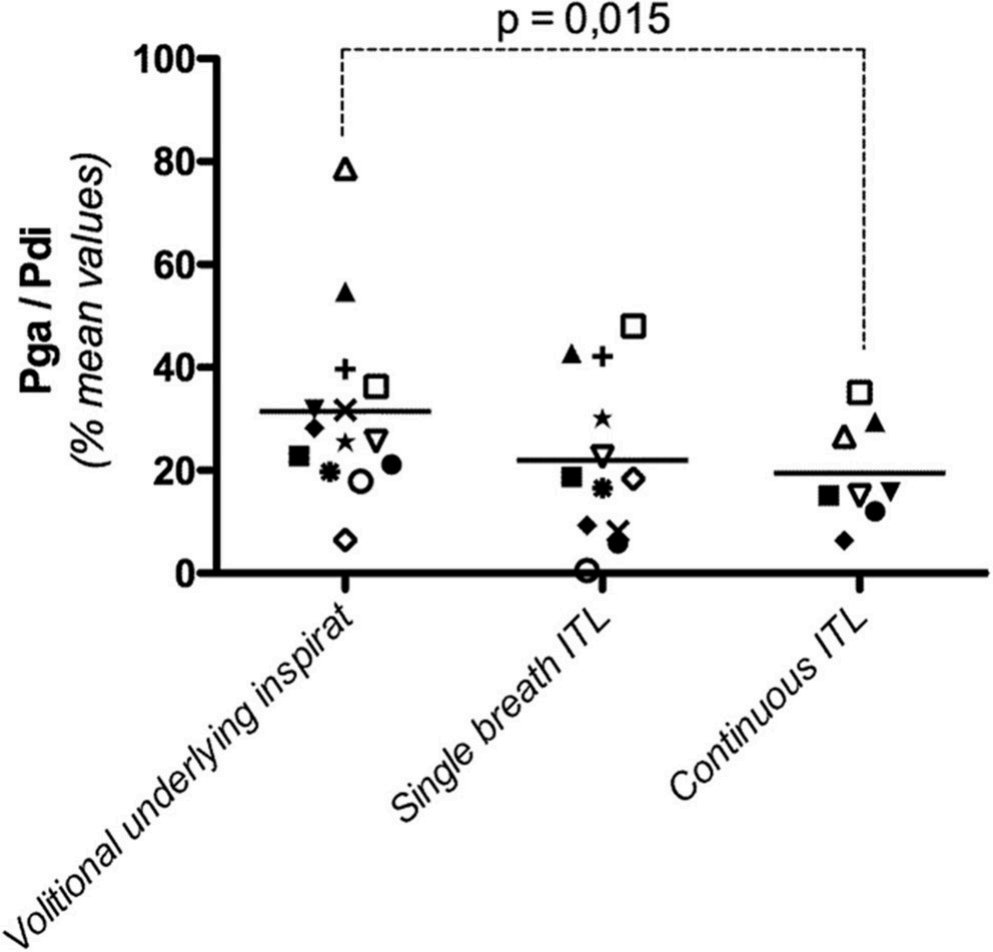
**Diaphragmatic contribution to the inspiratory effort produced to trigger the cervical magnetic stimulation (Pga/Pdi, with Pga: gastric pressure and Pdi: transdiaphragmatic pressure) during a voluntary effort, a non-volitional “single-breath” effort in response to the first presentation of an infinite resistance, and a non-volitional “continuous” effort, 10 breathing cycles after application of an inspiratory threshold load**. Each point represents the mean value of Pga/Pdi in one subject, irrespective of the intensity of the underlying effort. The horizontal lines depict the group mean value.

The overall Pga/Pdi ratio, corresponding to the underlying inspiratory effort in the 14 subjects for all levels of volitional effort, was 31 ± 17 (Table 2). Over the range of Pdi,vol studied, the Pga/Pdi ratio did not vary in 6 subjects, decreased significantly in 5 subjects and increased significantly in 3 subjects (Figure 3).

**Table 2:** **Slope of the gastric to transdiaphragmatic pressure ratio (Pga/Pdi) over the range of underlying Pdi studied during the three experimental conditions**.

	**Volitional underlying effort**	**Single breath inspiratory threshold loading**	**Sustained inspiratory threshold loading**
subject # 1	0.001205ns	−0.000618ns	−0.006111°
subject # 2	0.002875^*^	0.008295^*^	0.000253ns
subject # 3	−0.002505°		−0.000585ns
Subject # 4	0.004345^*^	−0.002961ns	−0.000197ns
Subject # 5	0.000041ns	0.000546ns	−0.000825ns
Subject # 6	0.003075ns	0.001612ns	0.008076^*^
Subject # 7	0.002590^*^		0.006949ns
Subject # 8	−0.000747ns	0.002988^*^	−0.005481°
Subject # 9	−0.000515ns	0.005537^*^	
Subject # 10	−0.004495°	−0.000438ns	
Subject # 11	0.003656^*^	−0.002304ns	
Subject # 12	0.004056ns	0.010400^*^	
Subject # 13	−0.002739°	0.001997^*^	
Subject # 14	−0.003207°	0.003598^*^	

The symbol “ns” indicates that Pga/Pdi did not significantly vary over the range of underlying Pdi studied, the symbol “^*^” indicates that it increased significantly, and the symbol “°” indicates that decreased significantly.

### Single-breath load twitch interpolation (“single-breath” procedure)

For technical reasons, the results of this procedure were available for only 12 subjects. The amplitude of the superimposed twitches also decreased linearly (Figure 2B) with the intensity of the underlying Pdi, according to y' = b'–a' x Pdi,single-breath (Equation 2).

With the same conventions as above, the slope “a” of Equation 2 was −1.5 ± 0.7 percent baseline.percent baseline^−1^, significantly different from the slope “a” of Equation 1 (*p* = 0.04). The Y-intercept of Equation 2 was 109 ± 13%, not significantly different from 100% (*p* = 0.14). In view of the steeper slope of the relationship, the extrapolation of Equation 2 through the abscissa yielded a Pdi,max value that was significantly lower than the measured Pdi,max (110 ± 40 vs. 141 ± 23 cm H_2_O, respectively, *p* = 0.027). In other words, the recruitment of phrenic motoneurons required to produce a given Pdi was greater during the “single breath” procedure than during the voluntary procedure (Figures 2A,B).

The overall value of the Pga/Pdi ratio, corresponding to the underlying inspiratory effort in the 12 subjects studied for all levels of volitional effort, was 22 ± 16 (Table 2). This value was not significantly different from that observed during the “vol” procedure (*p* = 0.13). Over the range of Pdi,single-breath studied, the Pga/Pdi ratio did not vary in 7 of the 12 subjects, and increased slightly but significantly in the remaining 5 subjects. Overall, no systematic difference was observed between “vol” and “single-breath” conditions in terms of the contribution of the diaphragm to the effort underlying (and triggering- the stimulation (Figure 3).

### Sustained inspiratory threshold loading twitch interpolation (“continuous” procedure)

For technical reasons, the results of this procedure were available for only 8 subjects (Figure 2C). In two subjects, no statistically significant relationship was demonstrated between Pdi,tw and Pdi,continuous. In the remaining 6 cases, a relationship was observed, but with a slope of −0.6 ± 0.4 percent baseline.percent baseline^−1^ that was significantly lower than the slope observed during the “vol” procedure (*p* = 0.017). In other words, the recruitment of phrenic motoneurons required to produce a given Pdi was lower during the “continuous” procedure than during the “single breath” procedure.

The overall value of the Pga/Pdi ratio in the 8 subjects studied was 19 ± 9%, significantly lower than that observed during the “vol” procedure (*p* = 0.015) (Figure 3). Over the range of Pdi,continuous studied, the contribution of the diaphragm to the inspiratory effort did not vary in 5 subjects, decreased significantly in 2 subjects and increased significantly in one subject.

## Discussion

This study shows that the relationship between the amplitude of the Pdi produced by a diaphragm twitch and the Pdi produced by the underlying diaphragm contraction varies according to the nature of the corresponding effort and therefore the nature of the corresponding neural command. Indeed, the slope of this relationship was significantly lower during inspiratory efforts induced under single-breath conditions by an inspiratory load than during inspiratory efforts produced voluntarily, despite a similar contribution of the diaphragm to the underlying effort (as assessed by the Pga/Pdi ratio). If twitch interpolation is interpreted as reflecting motoneuron recruitment (although such an interpretation must be proposed cautiously, see Gandevia, 2001; Herbert and Gandevia, 1999), this result would indicate that a given Pdi can be obtained with different phrenic motoneuron recruitment strategies. This study also shows, in line with the working hypothesis and with previous fMRI data (Raux et al., 2013), that the maintenance of an inspiratory threshold load over time is associated with modifications of the relationship between diaphragm twitch and the underlying diaphragm contraction and the contribution of the diaphragm to inspiration. These changes suggest a motor reorganization compatible with “diaphragm sparing.”

### Methodological considerations

A number of drawbacks can interfere with the construction of the twitch Pdi vs. “underlying Pdi” relationship and lead to misinterpretation of this relationship in terms of motoneuron activation (Gandevia, 2001). We tried to control for as many of these factors as possible. We used control twitches that were potentiated (Gandevia, 2001) and obtained during small underlying contractions to account for the corresponding changes in abdominal compliance (Hershenson et al., 1988) and for the sensitivity of Pdi to abdominal mechanical properties and configuration (Chen et al., 2000). We used supramaximal phrenic stimulation to avoid fluctuations in axonal activation. We limited the underlying contractions to a clearly submaximal range to minimize the nonlinearities that have been described during twitch interpolation procedures and modeling when underlying contractions become close to maximal (Herbert and Gandevia, 1999; Gandevia, 2001). Under these conditions, we observed linear relationships between twitch Pdi and underlying Pdi. We believe that these precautions reasonably allow us to compare the three types of underlying diaphragmatic contractions that we studied in terms of central drive to the diaphragm and the corresponding motoneuron recruitment. Nevertheless, antidromic collision and axon refractoriness are factors that can interfere with the interpretation of twitch interpolation, depending on stimulation characteristics and motor unit firing rates (Gandevia, 2001; Todd et al., 2003). We did not control for these factors, which could have been at least partly ensured by comparing the effects of underlying efforts on the output of phrenic stimulation and the output of transcranial magnetic stimulation (Similowski et al., 1996a; Todd et al., 2003). We therefore cannot exclude the possibility that antidromic collision and axon refractoriness differed between the three types of underlying diaphragmatic contractions studied and may therefore have contributed to the observed differences. Finally, twitch interpolation studies generally use isometric contractions upon which nerve stimulations or transcranial magnetic stimulations are superimposed (Merton, 1954; Gandevia, 2001). The complex geometry of the diaphragm implies that the isometric nature of a contraction is difficult to ascertain even when it is performed statically and isovolumetrically, i.e., against a closed airway. In the present study, the isovolumetric condition was respected in all experimental conditions, but phrenic stimulations were delivered when Pdi reached a preset threshold value during an inspiratory effort, namely during a dynamic contraction. Although the three study conditions were comparable in terms of the effort developed at the time of stimulation, the dynamic nature of the inspiratory effort may have involved variable degrees of “non-isometry” that could have influenced the twitch interpolation relationship. We believe that if such a factor was actually important, it would have introduced nonlinearities in this relationship, which were not observed.

### Neurophysiological considerations

Our findings during voluntary diaphragm contractions are in line with previous reports pertaining to diaphragm twitch interpolation (review in Gandevia, 2001), namely a linear Pdi,tw/Pdi,vol relationship, of which extrapolation through the abscissa provides a maximal Pdi,vol value matching the measured Pdi,max. During this type of effort (planned, self-timed, and voluntarily executed), the neural drive to breathe is most likely corticospinal without a bulbospinal relay or contribution. Indeed, although there is evidence for inhibitory connections from the cerebral cortex to the medulla in animals (Bassal et al., 1981; Bassal and Bianchi, 1981a,b; Orem and Netick, 1986; Orem, 1989) and in humans (McKay et al., 2008), there is no evidence for excitatory corticomedullary connections (Butler et al., 2014). In addition, performing a cortically prepared inspiration involves not only the production of force, but also control of inspiratory duration, which requires temporary inhibition of the medullary oscillator responsible for the bulbospinal respiratory drive. Several motor cortical areas are involved in the voluntary drive to breathe, the corticospinal output (Gandevia and Rothwell, 1987; Murphy et al., 1990; Colebatch et al., 1991; Similowski et al., 1996b; McKay et al., 2003) being modulated by cortico-cortical connections between the primary motor cortex and premotor areas (Macefield and Gandevia, 1991; Sharshar et al., 2004; Raux et al., 2007b, 2010; Laviolette et al., 2013). A different situation was observed during the “single-breath” and “continuous” parts of our experiments. Under “single-breath” conditions, inspiratory occlusions designed to increase inspiratory effort were superimposed on tidal breathing, and disrupted the automatic drive to breathe (bulbospinal output). As fMRI data have shown cortical activation in an identical context (Raux et al., 2013), the central nervous system input to phrenic motoneurons was therefore likely to be of mixed origin (bulbospinal and corticospinal). This was also the case for the “continuous” loading condition. To our knowledge, the recruitment of phrenic motoneurons from automatic bulbospinal drive has not been studied in humans with the twitch interpolation technique. The only available study on this subject was conducted in anesthetized rabbits (Ferguson, 1994). A linear relationship was found between the phrenic electroneurogram (increasing) and Pdi,tw (decreasing). This observation validates the twitch interpolation as a marker of phrenic spinal motoneurons recruitment, irrespective of the origin of the respiratory drive, allowing us to conclude that the very presence of a twitch interpolation pattern in response to “single-breath” occlusions is indicative of progressive motoneuron recruitment. Because the subjects were awake and because the duration of the inspiratory occlusions was longer than 100 ms (in order to reach the target Pdi), we postulate that this recruitment was most certainly the result of reinforced cortico-subcortical cooperation to overcome the load. A relationship between the intensity of an inspiratory load and the corresponding cortical activation has not been formally demonstrated. Nevertheless, pre-inspiratory potentials have incidentally been described as having a higher amplitude in response to a higher inspiratory load (Tremoureux et al., 2010), and a proportionality between the amplitude of movement-related motor cortical potentials and load that has been described during dynamic elbow flexions (de Morree et al., 2012). In contrast with the voluntary efforts of the “voluntary” experiments, the inspiratory efforts that triggered phrenic stimulation during the “single-breath” experiments were not planned in advance, but, in a sense, cued by the inspiratory occlusion. This aspect and the fact that a given underlying Pdi was associated with more twitch attenuation during the “single-breath” condition than during the “voluntary” condition (with similar Pga/Pdi ratios, Figure 3) indicate different central recruitment patterns [of note, the very possibility of generating the same Pdi with different levels of phrenic motoneuron recruitment serves as a reminder that the value of Pdi as a marker of diaphragm contraction may be altered by other respiratory muscles during synergistic efforts, because of imperfect pressure transmission across the contracted diaphragm (Laporta and Grassino, 1985)]. This is consistent with voluntary and cued actions being controlled by different brain systems —fronto-median cortex for voluntary actions (Deiber et al., 1999; Jenkins et al., 2000; Waszak et al., 2005), parietal and premotor cortices for stimulus-driven actions (Haggard, 2008)—although neurons in brain regions including the supplementary motor area discharge during both types of movements (Romo and Schultz, 1987; Kurata and Wise, 1988) and phenomenological evidence indicates an overlap and strong interdependency between the two systems (Hughes et al., 2011). The steeper slope of the twitch interpolation during the “single-breath” experiments in comparison with the “voluntary” experiments suggests that a larger part of the phrenic motoneuron pool was recruited during “single-breath” efforts to produce the same Pdi, which would be consistent with movement preparation resulting in a sort of “resource optimization” that is lacking in the case of sudden cued movement. In this regard, the “continuous” loading experiments were characterized by a much lower degree of diaphragm activation, as reflected by the reduced Pg/Pdi ratio (Figure 3) and the absence or quasi-absence of Pdi,tw attenuation with the underlying Pdi (Figure 2C), indicating that the contribution of the diaphragm to the inspiratory effort produced to overcome the threshold load decreased compared to “single-breath” experiments. These results suggest reorganization of the temporospatial distribution of the neural drive to inspiratory muscles (Butler, 2007; Butler et al., 2014), which fuels the notion that the changes in brain activation that have been described between single-breath and continuous loading at least partly correspond to motor phenomena (as opposed to mere sensory habituation). Cortical mechanisms might therefore play a role in the organization/reorganization of respiratory motoneuron output, possibly in addition to the role of a postulated premotoneuronal network of spinal interneurons (Butler et al., 2014).

In conclusion, this study confirms that the need to overcome a sustained inspiratory load in healthy humans is associated with reorganization of the central drive to breathe in general, and of the neural drive to the diaphragm in particular. These findings are in line with respiratory mechanics studies showing that the role of the various inspiratory muscle groups changes with loading (Aliverti et al., 1997; Yan and Kayser, 1997), apparently to reduce the burden of the diaphragm as a pressure generator and possibly to prevent diaphragm fatigue. They are also in line with data suggesting that inspiratory threshold loading can be associated with “diaphragm sparing” mechanisms in certain circumstances, for example during exercise (Jonville et al., 1985). Finally, they corroborate the observations previously made by our group with fMRI during single breath and sustained inspiratory threshold loading (Raux et al., 2013), showing that important reductions in cortical activation are observed both in premotor/motor and sensory cortical areas when inspiratory loading becomes sustained. Because the twitch interpolation phenomenon (Merton, 1954; Bellemare and Bigland-Ritchie, 1984; Gandevia, 2001) is a reflection of motoneuronal recruitment and therefore only involves motor activities (and no sensory activities), the present data confirm that the reductions in cortical activation observed when inspiratory loading becomes sustained have a motor component and are not only due to sensory habituation.

## Author contributions

MR contributed to study design, data acquisition, analysis and interpretation; drafted the manuscript; approved the final version of the manuscript. AD contributed to study design, data acquisition, analysis and interpretation; revised the initial draft of the manuscript; approved the final version of the manuscript. SR contributed to data acquisition, analysis and interpretation; approved the final version of the manuscript. CM contributed to data acquisition, analysis and interpretation; approved the final version of the manuscript. TS contributed to study design, data acquisition, analysis and interpretation; revised the initial draft of the manuscript; approved the final version of the manuscript. All authors agreed to be accountable for all aspects of the work in ensuring that questions related to the accuracy or integrity of any part of the work.

## Funding

This study was funded in part by “Association pour le Développement et l'Organisation de la Recherche En Pneumologie et sur le Sommeil (ADOREPS),” Paris, France a “Contrat de recherche triennal Legs Poix de la Chancellerie de l'Université de Paris,” Paris, France, the Fondation pour l'Avenir (AP-RMA-2015-037) and by the “Investissement, d'Avenir ANR-10-AIHU-06” programme of the French Government. Paris, France. Mathieu Raux was supported in part by a scholarship from the “Comité National Contre les Maladies Respiratoires,” Paris, France and in part by a scholarship from the “Fédération ANTADIR,” Paris, France.

### Conflict of interest statement

The authors declare that the research was conducted in the absence of any commercial or financial relationships that could be construed as a potential conflict of interest. The reviewer ARC and handling Editor declared their shared affiliation, and the handling Editor states that the process nevertheless met the standards of a fair and objective review.
